# It's the holes that matter

**DOI:** 10.18632/aging.101330

**Published:** 2017-11-15

**Authors:** Victoria C Cogger, Mashani Mohamad, David G Le Couteur

**Affiliations:** Ageing and Alzheimers Institute and ANZAC Research Institute, University of Sydney and Concord Hospital, Sydney, Australia

**Keywords:** endothelium, fenestrations, hyperinsulinemia, aging

Before circulating insulin can interact with membrane bound insulin receptors and trigger downstream signalling it must first cross the endothelium of the blood vessels in the target tissue. This transfer across the endothelium from the blood is recognised as a rate limiting step in insulin action in muscle and fat in humans [1], but the role of the liver endothelium in insulin uptake has not been examined previously. The paper by Mohamad et al. [2] explores the contribution of insulin transfer from the blood, across the liver sinusoidal endothelium and to the insulin receptors on the hepatocytes as a mechanism for the development of hyperinsulineamia and insulin resistance, as identified as a major risk factor for the development of age-related disease in humans [3].

The sinusoids, or blood vessels of the liver are lined by specialized endothelial cells that are very thin and perforated with transcellular holes or pores that traverse the entire cell from apical to basolateral membranes. These pores, known as fenestrations, have no diaph-ragm and are patent passages through the cell. The fenestrations provide efficient ultrafiltration of small material from the blood into the liver [4]. Coupled with very little extracellular matrix and a highly adapted hepatocyte membrane, uptake of substrates, such as nutrients, toxins and insulin into the liver for metabolism, detoxification and signalling is rapid and regularly overlooked. However, in older age, the morphology of the liver sinusoids and the endothelium changes significantly. The cells become thicker, and the diameter and number of fenestrations is reduced by up to 50 % (known as defenestration), there is extracellular matrix deposition and evidence of loss of hepatocyte microvilli. Collectively, these changes have been called pseudocapillarization. It has previously been shown that these changes reduce hepatocyte uptake of lipoproteins and some drugs [5, 6].

In the current work, the hepatic and systemic dis-position of insulin was explored in young and old animals and insulin resistance was confirmed to be present in the older animals. Critically, using multiple indicator techniques insulin transfer across the liver endothelium was shown to be significantly impaired. The 20 % reduction in insulin's volume of distribution in the liver was consistent with limited transfer across the sinusoidal endothelium and retention of insulin in the sinusoid. In concordance with these changes, there were very high circulating insulin levels indicative of both increased secretion (measured by circulating C-peptide levels) and impaired clearance. Despite normal glucose tolerance tests in the older animals, insulin resistance, as determined by the product of insulin and glucose (also known as HOMA-IR) was present. Of key importance, insulin and glucose uptake into muscle and fat was shown to be unchanged with age, suggesting age related insulin resistance was most likely being driven by impaired hepatic uptake and clearance. To explore insulin signalling in insulin treated livers phospho-proteomic and PCR studies were used, revealing significant impairment of insulin signalling pathways with no changes in gene expression- this is all highly consistent with the selective impact of age-related defenestration on hepatic insulin sensitivity.

A key strength of the study is the recognition of the multifactorial nature of aging and use of the acute model of defenestration, poloxamer 407 (P-407) [7], which was used to dissect the impact of fenestration loss on insulin uptake from the role of other potential age-related cellular processes. Again, multiple indicator dilution techniques showed significant retention of insulin in the sinusoids of the liver in P-407 treated animals versus young controls, this was supported by increased circulating insulin levels without increased insulin secretion (suggesting lack of adaptation). There were no changes in insulin uptake in the muscle or fat of the P407 treated animals, consistent with what was shown in the older animals. PCR and phospho-proteomics showed no change in genes expression, with impaired downstream insulin signalling in acutely defenestrated, insulin treated livers, again very much in line with diminished insulin action in the hepatocytes caused by defenestration.

This work suggests that defenestration and pseudo-capillarization of the liver sinusoidal endothelium seen in aging prevents the access of insulin to the insulin receptor on the hepatocyte membrane through impaired transfer across the endothelium. This results in hyper-insulinemia, impaired hepatic insulin signalling and insulin resistance. Further the work demonstrates, the liver endothelium does not provide a barrier for the uptake of insulin under normal conditions. In summary, patent fenestrations are required for hepatic insulin uptake, clearance, and signalling and loss of fenestra-tions is a probable causative mechanism for insulin resistance and diabetes seen with aging. This work provides evidence that maintaining the integrity of the liver sinusoidal endothelium into old age may prevent age-related insulin resistance and excitingly, introduces a novel therapeutic target.
